# Supplementary material for: Diclofenac Potentiates Sorafenib-Based Treatments of Hepatocellular Carcinoma by Enhancing Oxidative Stress
Source: Cancers (Basel). 2019 Sep 27;11(10):1453. doi: 10.3390/cancers11101453 (PMC6827164; doi:10.3390/cancers11101453)
Supplement: Supplementary file 1 [file cancers-11-01453-s001.pdf]

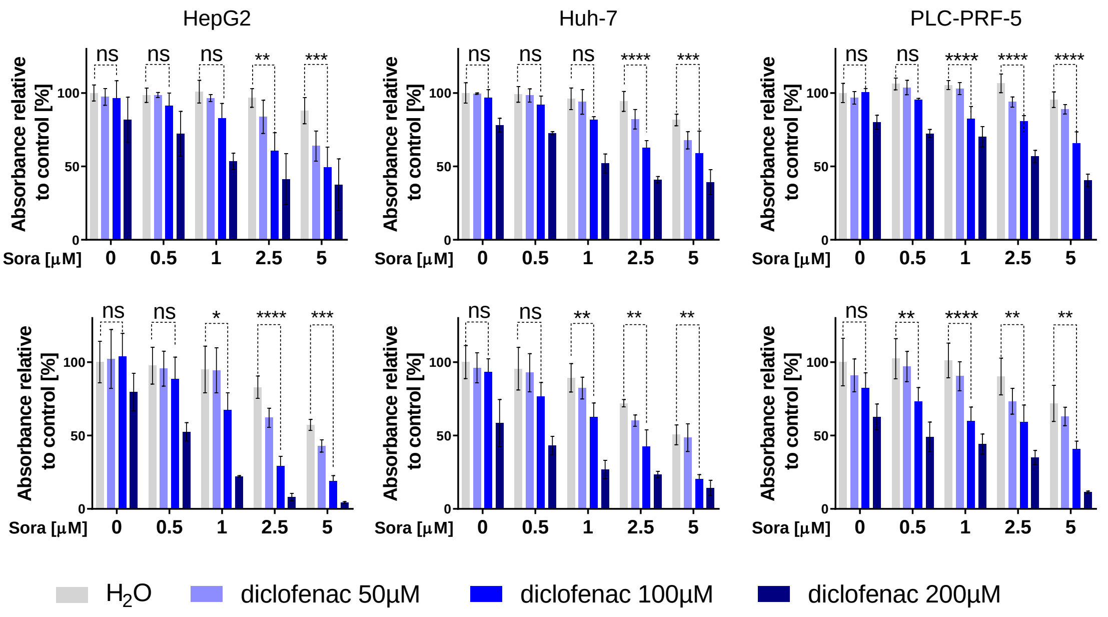

Figure S1. Dose and time effects of sorafenib and diclofenac on HCC cell growth. MTS proliferation assay of HepG2, HuH-7 and PLC-PRF-5 cell lines treated for 24 hours (upper panels) or 48 hours (lower panels) with indicated concentrations of sorafenib (Sora) or diclofenac or DMSO as control. Columns represent the means of three separate experiments run in triplicates. Mean of control group was fixed to 100 %. P value: * < 0.05, ** < 0.01, *** < 0.001, **** < 0.0001, ns: non-significant, two-way ANOVA with Sidak's multiple comparisons test.

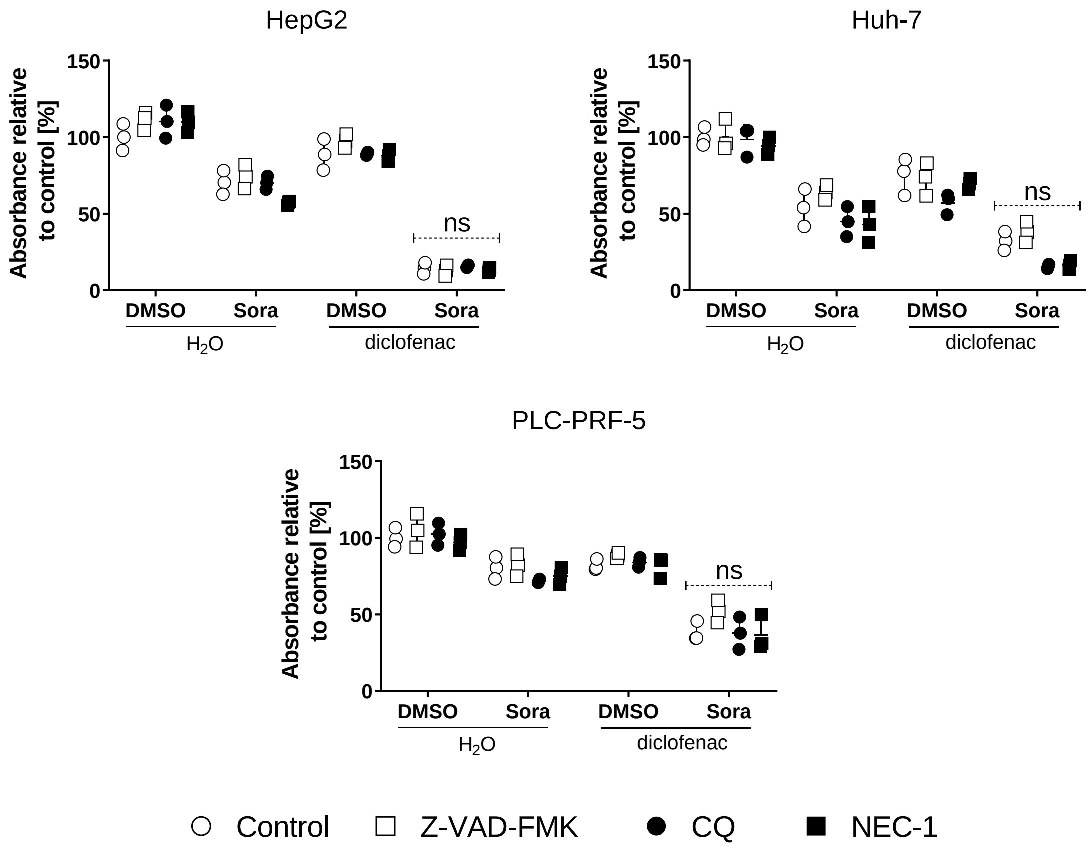

Figure S2. Effect of Z-VAD-FMK, chloroquine and necorstatin-1 on sorafenib/diclofenac-induced HCC cell death. MTS proliferation assay of HCC cell lines treated for 48 hours with sorafenib (Sora, 5 μM), diclofenac (100 μM), sorafenib and diclofenac or DMSO or H_2O as controls in presence or absence of Z-VAD-FMK (20 μM), chloroquine (CQ, 25 μM) or necrostatin-1 (NEC-1, 100 μM). Each dot represents the mean of a separate experiment run in triplicates. Mean of control group (DMSO/ H_2O) was fixed to 100 %. Ns non-significant as indicated, two-way ANOVA with Sidak's multiple comparisons test.
